# Epitranscriptomic N4-Acetylcytidine Profiling in CD4^+^ T Cells of Systemic Lupus Erythematosus

**DOI:** 10.3389/fcell.2020.00842

**Published:** 2020-08-28

**Authors:** Gangqiang Guo, Xinyu Shi, Huijing Wang, Lele Ye, Xinya Tong, Kejing Yan, Ning Ding, Chaosheng Chen, Huidi Zhang, Xiangyang Xue

**Affiliations:** ^1^School of Life Sciences and Technology, Tongji University, Shanghai, China; ^2^Department of Microbiology and Immunology, Institute of Molecular Virology and Immunology, Institute of Tropical Medicine, School of Basic Medical Sciences, Wenzhou Medical University, Wenzhou, China; ^3^Kidney Disease Center, The First Affiliated Hospital, College of Medicine, Zhejiang University, Hangzhou, China; ^4^Department of Gynecologic Oncology, Women’s Hospital, School of Medicine, Zhejiang University, Hangzhou, China; ^5^Department of Nephrology, The First Affiliated Hospital, Wenzhou Medical University, Wenzhou, China

**Keywords:** epitranscriptome, systemic lupus erythematosus, N4-acetylcytidine (ac^4^C), CD4^+^ T cells, NAT10

## Abstract

The emerging epitranscriptome plays an essential role in autoimmune disease. As a novel mRNA modification, N4-acetylcytidine (ac^4^C) could promote mRNA stability and translational efficiency. However, whether epigenetic mechanisms of RNA ac^4^C modification are involved in systemic lupus erythematosus (SLE) remains unclear. Herein, we detected eleven modifications in CD4^+^ T cells of SLE patients using mass spectrometry (LC-MS/MS). Furthermore, using samples from four CD4^+^ T cell pools, we identified lower modification of ac^4^C mRNA in SLE patients as compared to that in healthy controls (HCs). Meanwhile, significantly lower mRNA acetyltransferase NAT10 expression was detected in lupus CD4^+^ T cells by RT-qPCR. We then illustrated the transcriptome-wide ac^4^C profile in CD4^+^ T cells of SLE patients by ac^4^C-RIP-Seq and found ac^4^C distribution in mRNA transcripts to be highly conserved and enriched in mRNA coding sequence regions. Using bioinformatics analysis, the 3879 and 4073 ac^4^C hyper-acetylated and hypoacetylated peaks found in SLE samples, respectively, were found to be significantly involved in SLE-related function enrichments, including multiple metabolic and transcription-related processes, ROS-induced cellular signaling, apoptosis signaling, and NF-κB signaling. Moreover, we demonstrated the ac^4^C-modified regulatory network of gene biological functions in lupus CD4^+^ T cells. Notably, we determined that the 26 upregulated genes with hyperacetylation played essential roles in autoimmune diseases and disease-related processes. Additionally, the unique ac^4^C-related transcripts, including *USP18*, *GPX1*, and *RGL1*, regulate mRNA catabolic processes and translational initiation. Our study identified novel dysregulated ac^4^C mRNAs associated with critical immune and inflammatory responses, that have translational potential in lupus CD4^+^ T cells. Hence, our findings reveal transcriptional significance and potential therapeutic targets of mRNA ac^4^C modifications in SLE pathogenesis.

## Introduction

Systemic lupus erythematosus (SLE) is a heterogenous autoimmune disease with complicated clinical manifestations and high mortality, and is associated with development of autoantibodies and inflammatory responses resulting in damage to multiple organs and systems (Tsokos et al., 2016). Accumulating evidence indicates that the pathogenesis and etiology of this systemic disease are highly intricate, being influenced by several factors including genetic susceptibility, sex hormones, environmental risk factors, and immunological mechanisms (Rönnblom and Elkon, 2010; Liu and Davidson, 2012; Tsokos et al., 2016). Of these factors, imbalanced innate and adaptive immune responses, including immune cells (CD4^+^ T cells, B cells, and Th17 cells), cytokines (type I interferon and interleukin) and complement proteins contribute to inflammatory processes and tissue injury (Morel, 2017; Song et al., 2020). During this process, epigenetic abnormities can directly influence the autoimmune reaction by dysregulating immune cell function, particularly in CD4^+^ T lymphocytes (Wardowska, 2020).

Currently, our advanced understanding of the genetic mechanism underlying SLE indicated that epigenetic changes, such as disease-associated susceptibility gene regulation, non-coding RNA regulation, and post-transcriptional modifications (Meroni and Penatti, 2016; Mazzone et al., 2019), play important roles in SLE. For example, our previous study demonstrated that abnormally expressed microRNAs and non-coding circular RNAs (circRNAs) are involved in SLE pathogenesis and are potential diagnostic markers (Guo et al., 2018, 2019). Moreover, several studies have found that epigenetic changes, such as DNA and mRNA modifications are dysregulated and can alter gene expression in SLE CD4^+^ T cells (Zhang et al., 2013; Zhao et al., 2014, 2016). Recently, we also identified novel aberrant mRNA modification, 5-methylcytidine (m^5^C), linked to critical immune pathways in lupus CD4^+^ T cells (Guo et al., 2020).

Focusing on the epitranscriptome, mRNAs comprise a diverse array of posttranscriptional chemical modifications, which contribute to RNA structure and function alteration, as well as gene expression dysregulation (Peer et al., 2017; Zhao et al., 2017). Several regulatory complexities, including N6-methyladenosine (m^6^A), m^5^C, 5-hydroxymethylcytidine (hm^5^C), and N4-acetylcytidine (ac^4^C), have been identified as conserved in all domains of life (Delatte et al., 2016; Dominissini and Rechavi, 2017; Arango et al., 2018; Chen X. et al., 2019). Specifically, N-acetyltransferase 10 (NAT10) is reportedly the only known acetyltransferase for ac^4^C in human tRNAs and rRNAs (Chimnaronk et al., 2009; Ito et al., 2014). Moreover, a recent study identified ac^4^C as a novel mRNA modification catalyzed by NAT10, that promotes mRNA stability and translational efficiency (Arango et al., 2018). However, whether ac^4^C modification occurs in CD4^+^ T cells of SLE patients, and the specific correlation between mRNA ac^4^C modification and SLE pathogenesis is poorly understood.

In this study, using liquid chromatography-coupled tandem mass spectrometry (LC-MS/MS), we identified ac^4^C mRNA modification in lupus CD4^+^ T cells as a novel form of mRNA modification. Further, we utilized ac^4^C-RIP-Seq approach to measure global profile of ac^4^C modification in CD4^+^ T cells from SLE patients and healthy controls (HCs). Our results demonstrated prominent variation of ac^4^C patterns in SLE and showed that the modified mRNA was significantly enriched in disease-associated functional pathways. Taken together, we demonstrate that acetylation cytidine mRNA, a new class of the epitranscriptome in SLE, can potentially be a promising diagnostic marker and therapeutic target.

## Materials and Methods

### Patient Information

In this study, we recruited 39 patients with SLE from the First Affiliated Hospital of Wenzhou Medical University, between December 2019 and June 2020. All SLE patients fulfilled the diagnostic criteria of the American College of Rheumatology (ACR). In addition, clinical data were assessed at the time of blood collection. A total of 51 age- and sex-matched HCs without renal failure, heart failure, or arthralgia, and free from any inflammatory conditions, were recruited from the same hospital. For mRNA liquid chromatography-coupled triple quadrupole tandem mass spectrometry (LC-MS/MS) and RNA ac^4^C-RIP-Seq, 10 SLE patients and 18 HCs were divided into four groups as our previous report (Guo et al., 2020). For each SLE group, five SLE samples were mixed and sequenced; similarly, each control group comprised nine mixed and sequenced. To quantify NAT10 expression levels, samples from 20 SLE patients and 21 HCs were analyzed via RT-qPCR. Meanwhile, to validate genes with dys-acetylated sites, samples from 9 SLE patients and 12 HCs were utilized for ac^4^C-RIP-qPCR. This study was approved by the Medical Ethical Committees of the First Affiliated Hospital of Wenzhou Medical University (No. 2019121). All participants provided written informed consent.

### CD4^+^ T Cell Isolation and RNA Extraction

Peripheral blood mononuclear cell (PBMCs) were isolated from HCs and SLE patients using human peripheral blood lymphocyte separation medium (Tianjin HaoYang Biological Manufacture, Tianjin, China) within 6 h of sample collection. CD4^+^ T cells were then purified following immunomagnetic separation using a human CD4^+^ T cell isolation kit (BD Biosciences, San Jose, CA, United States) according to the manufacturer’s instructions. For RNA purification, the isolated human CD4^+^ T cells were lysed in TRIzol reagent (Invitrogen Life Technologies, Grand Island, NY, United States). The extracted RNA was further digested by DNase I (Invitrogen, Waltham, MA, United States) to remove residual DNA, and subsequently separated from each sample using TRIzol reagent/RNeasy Mini kit (Qiagen, Hilden, Germany). Total extracted RNA was stored for use at −80°C.

### Liquid Chromatography-Coupled Tandem Mass Spectrometry (LC-MS/MS)

Total RNA was quantified and qualified using NanoDrop ND-1000 (Thermo Fisher Scientific Inc., Waltham, MA, United States) and Agilent 2100 Bioanalyzer (Agilent Technologies, Palo Alto, CA, United States). mRNA was isolated from total RNA using the NEBNext Poly(A) mRNA Magnetic Isolation Module (E7490; New England Biolabs, Inc., Ipswich, MA, United States) according to the manufacturer’s protocol. The detailed experimental methods and analyses monitoring peak information of utilized modified nucleosides were similar to those used in our recent study (Guo et al., 2020).

### Reverse Transcription Quantitative Polymerase Chain Reaction (RT-qPCR)

The NAT10 expression levels in CD4^+^ T cells of 41 individuals (21 HCs and 20 SLE patients) were measured by RT-qPCR using an Applied Biosystems QuantStudio^TM^ 3 real-time PCR instrument (Thermo Fisher Scientific Inc.). All RT-qPCR experiments were performed using the QuantiNova SYBR Green PCR kit (Qiagen, Hilden, Germany). For each reaction, 1 μL of diluted cDNA was mixed with 10 μL of 2 × SYBR Green PCR Master Mix. A final volume of 20 μL was achieved by the addition of 1.4 μL forward and reverse primers (10 μmol). The conditions for PCR amplification were as follows: 95°C for 2 min, followed by 40 cycles each of 95°C for 5 s and 60°C for 10 s. All samples were tested in triplicates. The data were analyzed by the comparative threshold cycle (*C*t) method. *GAPDH* was utilized as the control, and the relative quantification of *NAT10* in CD4^+^ T cells was calculated using the following equation: amount of target = 2^–Δ*Ct*^, where Δ*C*t = Ct_*NAT*10_ − Ct_*GAPDH*_. The gene-specific primers of *NAT10* and *GAPDH* used for RT-qPCR were listed in Supplementary Table S1.

### Acetylated RNA Immunoprecipitation Sequencing (ac^4^C-RIP-Seq) and Bioinformatics Analyses

Briefly, mRNA enrichment from the total RNA was performed using the Dynabeads^TM^ mRNA Purification kit (invitrogen) as per the manufacturer’s instructions and then subjected to immunoprecipitation (IP). RNA was randomly fragmented to ∼200 nt by RNA fragmentation reagents. The RNA fragments were incubated with the ac^4^C antibody (ab252215, Abcam, Cambridge, United Kingdom) by rotating at 4°C for 2 h. The mixture was then immunoprecipitated by incubation with Protein A/G beads (Thermo Fisher Scientific, MA, United States) at 4°C for an additional 2 h. Subsequently, the bound RNA were eluted from the beads and purified with Trizol reagent (Thermo Fisher) according to the manufacturer’s instructions. RNA libraries for IP and input samples were then constructed with NEBNext^®^ Ultra II Directional RNA Library Prep kit (New England Biolabs, Inc., United States) according to the manufacturer’s instructions. Libraries were qualified using Agilent 2100 bioanalyzer and then subjected to 150-bp paired-end sequencing on an Illumina HiSeq 4000 sequencer (Illumina, Inc., San Diego, CA, United States), and were controlled by Q30. After 3′ adaptor-trimming and removing low quality reads using cutadapt software (v1.9.3), clean reads of all libraries were aligned to the reference genome (HG19) by Hisat2 software (v2.0.4) (Kim et al., 2015). Acetylated sites on RNAs (peaks) were identified by MACS software (Feng et al., 2012). Differentially acetylated peaks with a fold change cutoff > 2 and *P* < 0.0001 were identified by diffReps (Shen et al., 2013). Identified ac^4^C peaks were subjected to motif enrichment analysis by DREME (Bailey, 2011), and metagene ac^4^C distribution was characterized by R package MetaPlotR (Olarerin-George and Jaffrey, 2017). Moreover, NGS for mRNA profiling was also performed using input samples. Differentially expressed genes (DEGs) were defined with a fold change ≥ 1.5, and *P* < 0.05. The detailed analyses have been reported in our previous study (Guo et al., 2018).

To further explore the essential role of ac^4^C mRNA modification in SLE, DEGs were separated into four groups based on whether the modifications resulted in hyper- or hypoacetylation of ac^4^C, and based on the up- or down-regulation of gene expression (fold change > 1.5; *P* < 0.05). The signaling network in SLE was assessed with Thomson Reuters database^1^ (Schuierer et al., 2010). GO analysis was performed by Metascape^2^ and DAVID (version 6.8^3^). GO terms with *P* < 0.05 were considered statistically significant. The PPI network analysis of DEGs from the four groups mentioned above for assessing generation, pathway enrichment, and visualization were performed as previously described (Han et al., 2020).

### ac^4^C-RIP -qPCR

Twenty micrograms of total RNA was extracted from 9 SLE patients and 12 HCs and subjected to ac^4^C-RIP, as described above. The mRNA enriched with ac^4^C in each sample was determined via RT-qPCR, and quantified by normalizing to nine-fold input. The gene-specific qPCR primer sequences are presented in Supplementary Table S1.

### Statistical Analysis

Statistical analysis was performed using SPSS 22.0 software (IBM, Armonk, NY, United States). To assess the significant difference between two groups, a two-sided independent-samples *t*-test or two-sided Mann–Whitney *U*-test were performed as indicated. A two-sided *P*-value < 0.05 was regarded as a statistically significant difference.

## Results

### Eleven mRNA Modifications of CD4^+^ T Cells Between SLE Patients and HCs

**FIGURE 1:**
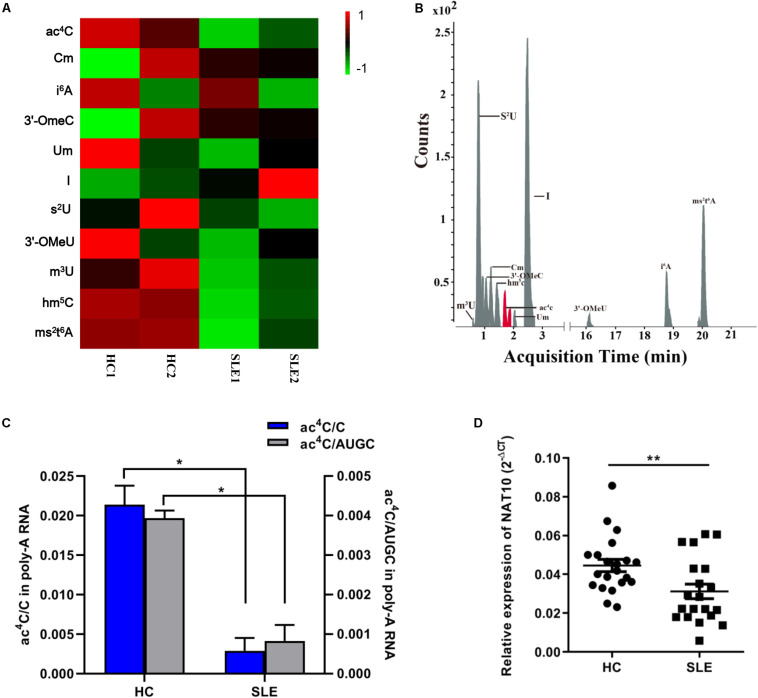
Overview of mRNA ac^4^C acetylation mapping between systemic lupus erythematosus (SLE) patients and healthy controls (HCs). **(A)** Heatmap of normalized abundance (modification/canonical nucleotide) of 11 mRNA modification forms detected through LC-MS/MS in CD4^+^ T cells from SLE patients and HCs. Green indicates a low *z*-score whereas red indicates a high *z*-score. **(B)**. LC-MS/MS extracted ion chromatograms of modified mRNA in SLE patients and HCs. ac^4^C, N4-acetylcytidine; Cm, 2′-*O*-methylcytidine; i^6^A, N6-isopentenyladenosine; 3′-OmeC, 3′-*O*-methylcytidine; Um, 2′-*O*-methyluridine; I, inosine; s^2^U, 2-thiouridine; 3′-OMeA, 3′-*O*-methyladenosine; m^3^U, 3-methyluridine; hm^5^C, 5-hydroxymethylcytidine; ms^2^t^6^A, 2-methylthio-N6-threonylcarbamoyladenosine. **(C)** Comparation of calibrated ac^4^C/C and ac^4^C/AUGC levels in poly(A) RNA between HCs and SLE. Bars show the mean with SD of individual biological replicates (*n* = 2). *P < 0.05. **(D)** NAT10 mRNA expression level in CD4^+^ T cells from HCs (*n* = 21) and SLE patients (*n* = 20). Compared to HCs, NAT10 was downregulated in SLE patients. Data are presented as 2^–Δ*Ct*^ relative to GAPDH expression (mean with SEM; **P < 0.01).

We combined equal amounts of mRNA from the CD4^+^ T cells of five SLE patients or nine HCs, into one pool for each group, thus obtaining four separate CD4^+^ T cell pools as previously described (Zhao et al., 2014). We first utilized LC-MS/MS to generate mRNA modification for the four separate pools and identified mRNA modification in SLE patients and HCs. A total of 11 modifications, previously identified in mRNA, were detected in the two groups including ac^4^C, 2′-*O*-methylcytidine (Cm) and hm^5^C (Figures 1A,B). Compared with that in HCs, the inosine (I) level in CD4^+^ T cells of SLE was slightly elevated whereas that of other modifications were obviously decreased (Figure 1A). Based on a previous report (Arango et al., 2018), ac^4^C in eukaryotes functions in the regulation of gene expression by enhancing stability and translation of mRNA, potentially at the level of decoding efficiency. Therefore, we also evaluated the ac^4^C level in overall mRNA of patients with SLE. Compared to those of HCs, both the ac^4^C/C and ac^4^C/AUGC levels in poly (A) RNA of CD4^+^ T cells were significantly lower in SLE patients (*P* < 0.05; Figure 1C). Moreover, compared with that in HCs, the expression level of acetyltransferase NAT10 was markedly decreased in CD4^+^ T cells from SLE patients (*P* < 0.001; Figure 1D). These results suggested that NAT10 expression might be associated with the alternation of global ac^4^C levels in SLE.

### Transcriptome-Wide Global ac^4^C Modification in SLE Patients and HCs

**FIGURE 2:**
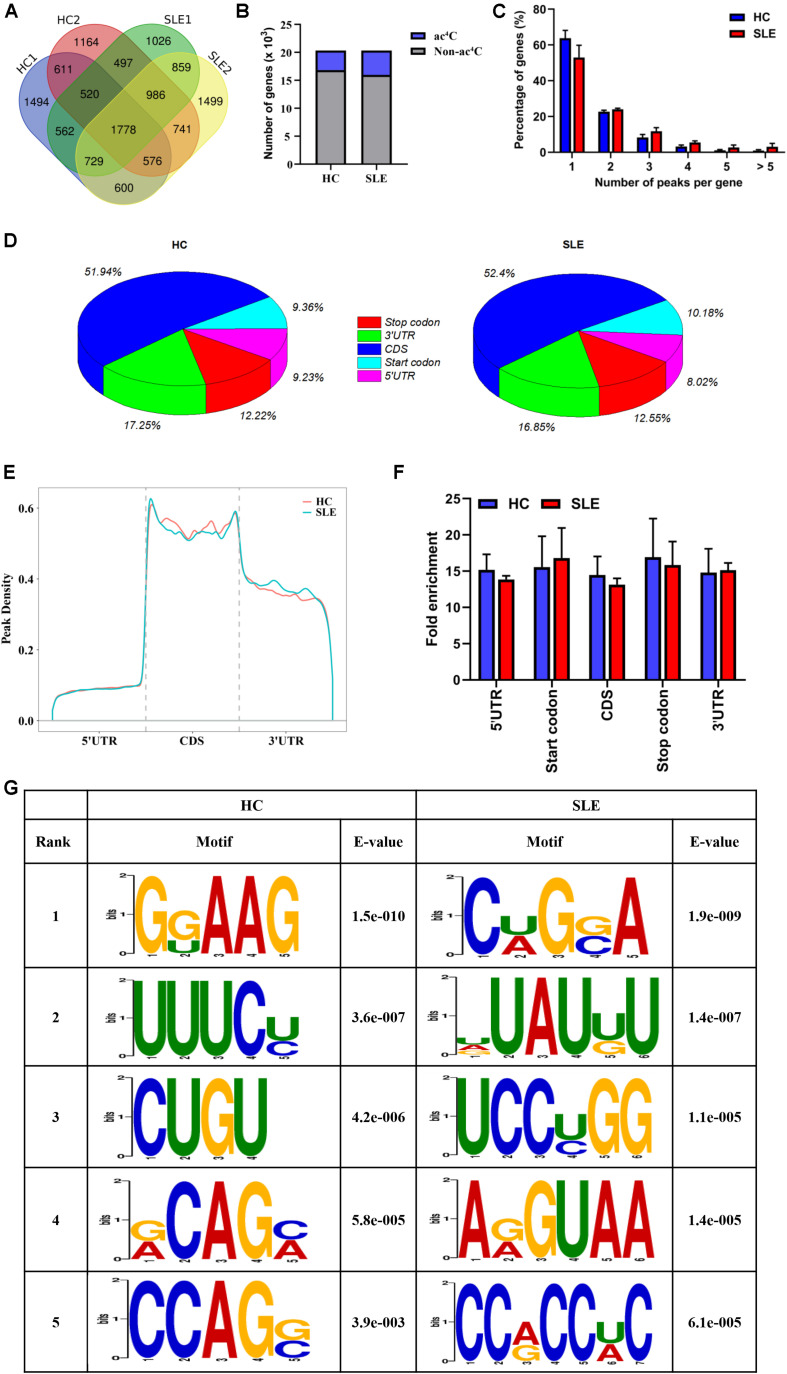
Transcriptome-wide ac^4^C-RIPSeq and distribution of ac^4^C peaks. **(A)** Venn diagram of ac^4^C acetylated genes in HCs and SLE patients. The numbers of HC-unique, SLE-unique, and common ac^4^C modified genes are shown. **(B)** Bar charts indicating the number of genes with no (gray) or at least one significant (blue) ac^4^C peak at SLE group or HC group. **(C)** Proportion of genes harboring different numbers of ac^4^C peaks in the two groups. The majority of transcripts harbor only one or two ac^4^C peaks per gene in HC and SLE groups. **(D)** Pie charts show ac^4^C peaks distribution in different gene context in HCs and SLE patients. The averages of percentages of mRNA ac^4^C peaks within 5′UTR, StartC, CDS, StopC, and 3′UTR regions in transcriptomes are shown. **(E)** Distribution of ac^4^C peaks along mRNA transcripts. The moving averages of percentages of mRNA ac^4^C peaks are shown. **(F)** Statistics of fold enrichment of ac^4^C peaks in five segments in the two groups. Error bars represent the mean with SD. **(G)** Sequence logo showing the top five differential mode motifs enriched across changed ac^4^C peaks identified from HCs and SLE patients.

To observe the global profile of transcriptome-wide ac^4^C modification, we further performed ac^4^C-RIP-Seq on the mRNA samples extracted from CD4^+^ T cell pools of SLE patients and HCs, which were the same samples as those used for the LC-MS/MS analyses. The number of overlaps and differences in ac^4^C-modified transcripts among the four separate biological pools are displayed in a Venn diagram (Figure 2A). A total of 3485 and 4352 acetylated genes transcripts were detected in HC replicates and SLE patients, respectively. Among all ac^4^C-represented transcripts within the two groups, only 1778 ac^4^C-modified mRNAs were identified. Compared with HCs, the SLE group lost 3269 representative acetylated gene transcripts and gained 3384 new ac^4^C-modified mRNAs. When compared with that in HCs, the number of ac^4^C-modified mRNAs were increased in CD4^+^ T cells from SLE patients (Figure 2B).

Next, we further analyzed the distribution of ac^4^C peaks per gene between the two groups. Intriguingly, in the SLE and HC groups, the majority of ac^4^C-modified transcripts were observed to have one or two ac^4^C peak(s) per gene (77.06 and 86.53%, respectively), while a very small number of transcripts contained three or more ac^4^C peaks (Figure 2C), which is similar to the trend of m^6^A characteristics previously reported in abnormal mouse livers (Luo et al., 2019). Furthermore, the proportion of ac^4^C-modified transcripts harboring only one ac^4^C peak was lower in CD4^+^ T cells of SLE groups (63.87% vs. 53.03%) than in HCs; whereas, the percentage of transcripts harboring two or more ac^4^C peaks was higher. By analyzing the distribution profile of ac^4^C peaks within mRNAs of SLE and HC samples, ac^4^C peaks were divided into five segments, according to their location in RNA transcripts (Figure 2D). Notably, the ac^4^C peaks were primarily enriched in coding sequences (CDS), stop codons (StopC), and 3′-untranslated regions (3′UTR) in both SLE and HC groups (Figure 2E), which differs from previous observations in HeLa cells (Arango et al., 2018). Compared with those of HCs, the fold enrichment of ac^4^C peaks showed no distinct difference in the five regions of lupus CD4^+^ T cells (Figure 2F). Furthermore, we investigated ac^4^C-modified peaks in the consensus sequences shared between the SLE and HC groups, using HOMER software. The ac^4^C peaks were characterized by the GRAAG motif in HCs, and CRGRA motif in SLE patients, of which, the top five ac^4^C peak motifs in SLE patients comprised CCRCCR (Figure 2G), which is similar to the results previously observed in HeLa cells (Arango et al., 2018).

### Functional Pathways of Differentially Acetylated Peaks Between SLE Patients and HCs

**FIGURE 3:**
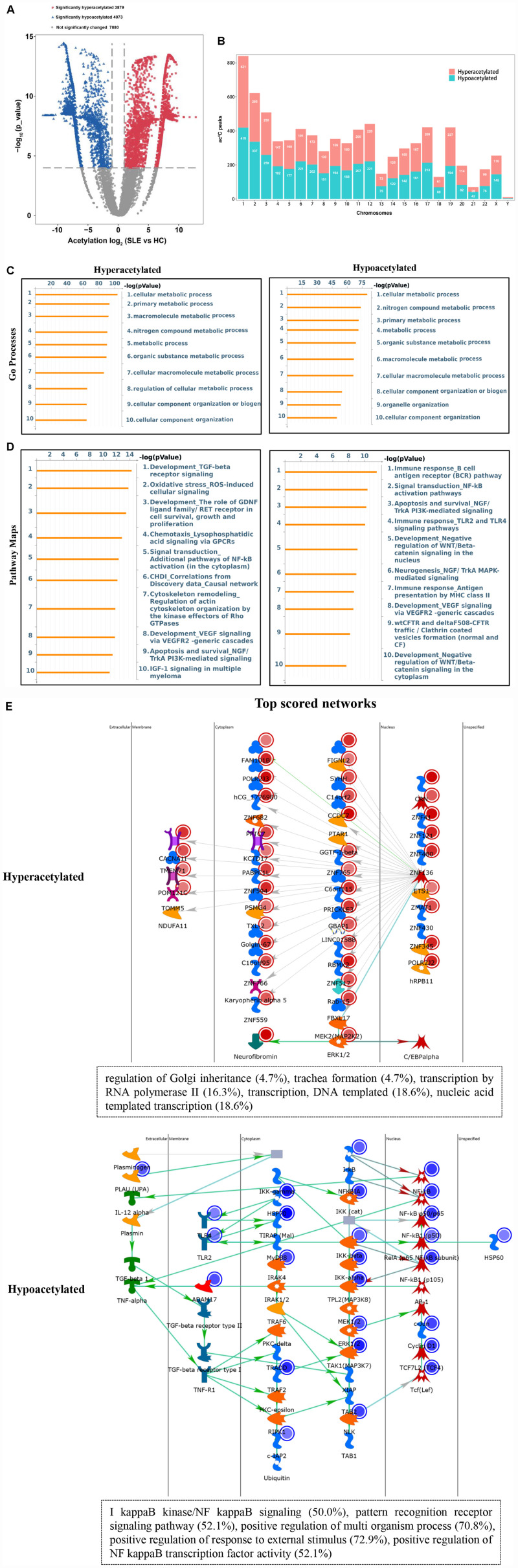
Pathway analysis of differentially acetylated genes. **(A)** Volcano plots display the differential ac^4^C peaks with statistical significance (fold change > 2; *P* < 0.0001). Blue points indicate significantly hypoacetylated transcripts and red points indicate significantly hyperacetylated transcripts. **(B)** The distributions of altered ac^4^C peaks in chromosomes of SLE patients. **(C)** Gene Ontology (GO) process analysis of differentially acetylated genes. Left panel represents hyperacetylated transcripts and right panel represents hypoacetylated transcripts. **(D)** Pathway Maps analysis of differentially acetylated genes. Left panel represents hyperacetylated transcripts and right panel represents hypoacetylated transcripts. **(E)** Top scored networks analysis of differentially acetylated genes. Top panel represents hyperacetylated transcripts and bottom panel represents hypoacetylated transcripts.

To explore the potential biological function of ac^4^C modification in SLE, the abundance of the ac^4^C-modified peaks between SLE and HC samples was compared. Among all ac^4^C peaks detected in both the groups, a total of 7952 significantly dysregulated acetylated peaks were selected for sequential study (fold change > 2; *P* < 0.0001). A total of 3879 ac^4^C hyperacetylated, and 4073 ac^4^C hypoacetylated peaks were detected in SLE patients and compared with those in HCs (Figure 3A). The distribution of altered ac^4^C peaks in the two groups revealed that the differentially ac^4^C-modified peaks were transcribed from all chromosomes, particularly chr1, chr2, and chr3 (Figure 3B). We further performed Pathway analysis in dys-acetylated peaks (hypo- or hyper-) from CD4^+^ T cells of SLE patients using GO, KEGG pathway, and Thomson Reuters database analysis. GO process results showed that both the dys-acetylated peaks were significantly enriched in the regulation of metabolic processes, including cellular metabolic processes, primary metabolic processes, and nitrogen compound metabolic processes (Figure 3C). In the Pathway map analysis, the hyperacetylated peaks were markedly associated with the modulation of TGF-β receptor signaling, oxidative stress ROS-induced cellular signaling, and signal transduction pathways involving NF-κB activation (Figure 3D). Moreover, the hypoacetylated peaks involved multiple biological pathways, including immune response B cell antigen receptor (BCR) pathway, signal transduction NF-κB activation pathway, and antigen presentation by MHC II (Figure 3D). Furthermore, the top scored network analyses of hyperacetylated peaks were found to be involved in translational processes including nucleic acid-templated transcription (18.6%), DNA-templated transcription (18.6%), and transcription by RNA polymerase II (16.3%) (Figure 3E and Supplementary Table S2). The top scored networks of hypoacetylated peaks were enriched in positive regulation of multi-organism processes (70.8%), positive regulation of responses to external stimulus (72.9%), positive regulation of NF-κB transcription factor activity (52.1%), and I-κB kinase/NF-κB signaling (50.0%) (Figure 3E and Supplementary Table S2), which agrees with the pathway map analysis. Notably, we selected the top scored networks for the hypoacetylated peaks (including ATP2C1, IKBKB, NFKB1, MAPK3, and TLR2) that were enriched in positive regulation of NF-κB transcription factor activity and I-κB kinase/NF κB signaling, and quantified the acetylation levels of these peaks by ac^4^C-RIP-qPCR. All of these peaks in CD4^+^ T cells of SLE patients were found to be hypoacetylated when compared to those in HCs. Particularly, IKBKB, NFKB1, and MAPK3 in CD4^+^ T cells of SLE patients were significantly hypoacetylated, which was consistent with the results of the ac4C-RIP profiles (Supplementary Figure S1). Taken together, it indicated that these differentially acetylated peaks are significantly enriched in crucial disease-related functional pathways.

### Functional Enrichment of Differentially Expressed Genes (DEGs) Between SLE Patients and HCs

To obtain the disease-related regulatory pathway of altered genes in lupus CD4^+^ T cells, we further investigated the functional enrichment of DEGs using Pathway maps, and Process Networks analyses. By analyzing the abnormally expressed mRNA profiles of the CD4^+^ T cells from SLE and HC groups, a total of 674 upregulated mRNAs, and 292 downregulated mRNAs were identified (fold change > 1.5; *P* < 0.05; Supplementary Figure S2A). Similar to previous reports on peripheral blood mononuclear cells (PBMCs) of SLE patients (Guo et al., 2018), pathway analysis showed that DEGs were associated with immune-related pathways (NETosis in SLE, antigen presentation by MHC class I and class II, IL-5 signaling via JAK/STAT, and IFN-alpha/beta signaling via MAPKs) and inflammatory processes (neutrophil activation, phagocytosis, chemotaxis, complement system) (Supplementary Figures S2B,C). Consistent with differentially acetylated peaks, DEGs were also found to be relevant to several functional pathways including signal transduction, antigen presentation by MHC II, and positive regulation of NF-kB transcription factor activity.

### The Association Between mRNA Expression and ac^4^C-Modified Transcripts in SLE Patients

**FIGURE 4:**
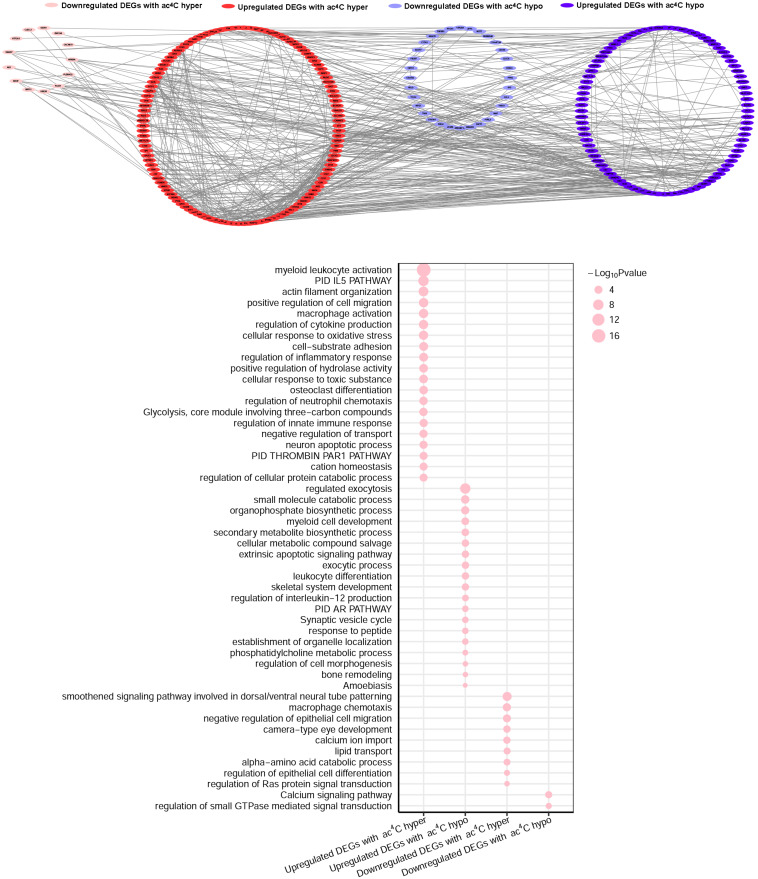
Gene Ontology enrichment map for ac^4^C-modified DEGs. Groups of upregulated DEGs with ac^4^C hyperacetylation (red), downregulated DEGs with ac^4^C hyper (pink), upregulated DEGs with ac^4^C hypo (dark blue), and downregulated DEGs with ac^4^C hypo (light blue) are marked in the network (*P* < 0.01; for details, see Supplementary Tables S3, S4) **(top panel)**. Bubble diagram of GO biological process categories enriched for DEGs with ac^4^C hyper- or hypo-acetylation. The pink circle represents the –log^10^ *P*-value **(down panel)**.

We then investigated whether ac^4^C-modified transcripts were associated with gene expression levels. To this end, we combined the RNA-Seq data of altered genes with the RNA ac^4^C-RIP-Seq data for the ac^4^C hyperacetylation or hypoacetylation, and found that approximately 75% (249/329) of the genes with ac^4^C hyperacetylation or hypoacetylation exhibited upregulated expression levels. Next, we defined all abnormally expressed/modified genes into four sets: upregulated genes with ac^4^C hypoacetylation (hypo-up set, *n* = 109), upregulated genes with ac^4^C hyperacetylation (hyper-up set, *n* = 140), downregulated genes with ac^4^C hypoacetylation (hypo-down set, *n* = 54), and downregulated genes with ac^4^C hyperacetylation (hyper-down set, *n* = 26) (Supplementary Figure S3). However, the data did not show a linear relationship between acetylation and expression (*R* = 0.139). To further investigate the biological and molecular function of ac^4^C-modified DEGs in SLE patients, comprehensive functional enrichment analysis was carried out. The PPI network and GO analyses of processes associated with the four gene sets apparently linked multiple functional processes and pathways (Figure 4 and Supplementary Tables S3, S4). We found that the genes of the hyper-down set were associated with macrophage chemotaxis, negative regulation of epithelial cell migration, and amino acid catabolic process; whereas, genes in the hypo-down set were associated with calcium signaling pathway and regulation of small GTPase mediated signal transduction. Genes in the hypo-up set were associated with multiple metabolic activities, including regulated exocytosis, small molecule catabolic processes, and secondary metabolite biosynthetic processes. Meanwhile, the genes included in the hyper-up set were significantly associated with macrophage activity and cytokine-mediated signaling pathway, thereby contributing to the recruitment and activation of inflammatory cells and immune responses. These results suggested that DEGs with ac^4^C modification affect key functional processes and pathways that related to immunology.

### ac^4^C Modification Participates in the Mechanism Underlying SLE Pathogenesis by Regulating Immune and Inflammatory Responses and Transcription-Related Processes

**FIGURE 5:**
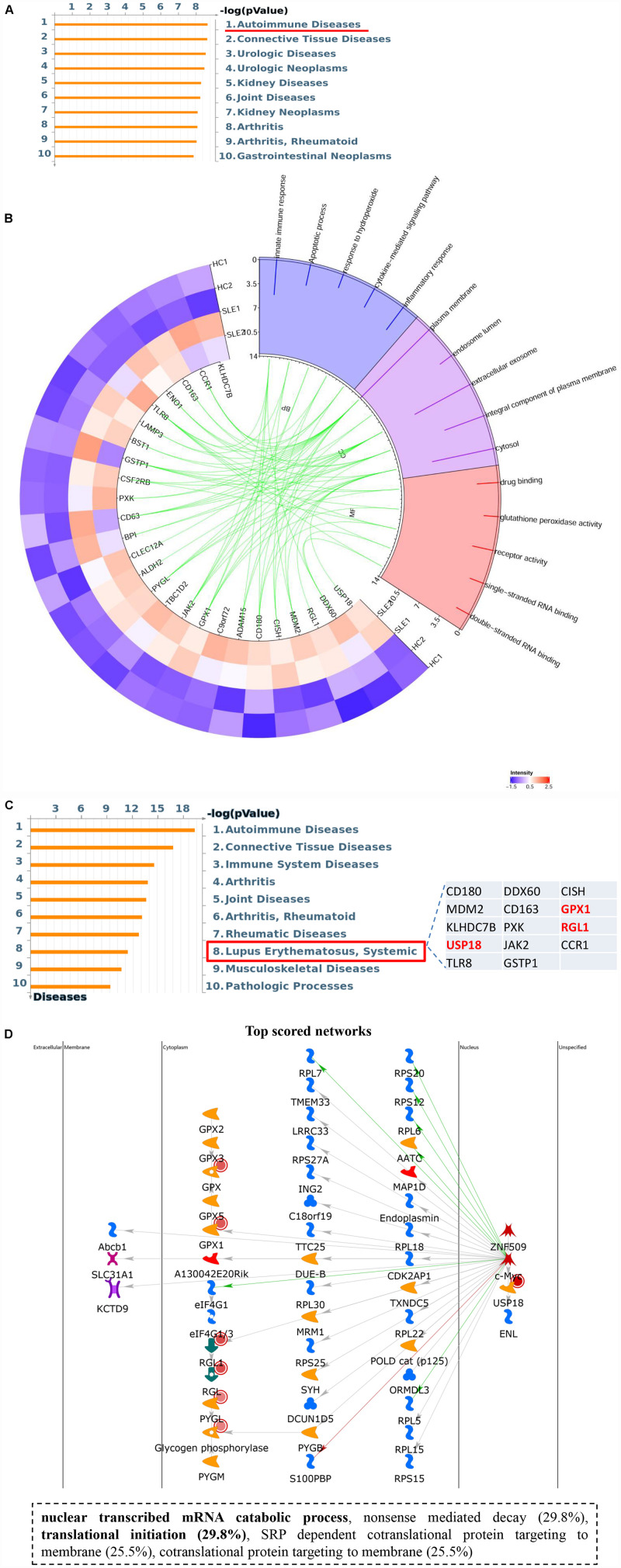
Upregulated gene set with ac^4^C hyperacetylation associated with SLE. **(A)** Pathway enriched analyses of upregulated DEGs with ac^4^C hyper. Twenty-six genes of upregulated DEGs with ac^4^C hyper-associated with autoimmune disease. **(B)** GO analysis of 26 upregulated DEGs with ac^4^C hyper. **(C)** Disease marker analysis of 26 upregulated DEGs with ac^4^C hyper. Fourteen of 26 genes of upregulated DEGs with ac^4^C hype associated with SLE. **(D)** The top scored networks analysis of 26 upregulated DEGs with ac^4^C hyper. GPX1, RGL1, and USP18 were involved in the top scored networks.

Intriguingly, hyper-up DEGs were found to participate in diverse important SLE-related biological processes, including inflammatory and immune responses, as well as regulation of cytokines and cellular response to oxidative stress. Moreover, the pathway enrichment results for the four DEGs sets identified a special cluster of DEGs, which were highly correlated with autoimmune diseases in the hyper-up set, consisting of 26 specific genes (Figure 5A and Supplementary Table S5). Therefore, we further selected these specific 26 upregulated DEGs with ac^4^C hyperacetylation to pursue the potential effects by GO analyses in CD4^+^ T cells of SLE patients. As shown in Figure 5B, the selected genes are notably abundant in vital functions including biological processes (innate immune response, inflammatory response, and cytokine-mediated signaling pathway), cellular components (plasma membrane and extracellular exosome), and molecular functions (drug binding and receptor activity). Particularly in biological process analyses, the results are consistent with those of the functional enrichment results of DEGs in SLE, which indicated that the 26 ac^4^C modified genes are highly relevant to the pathogenesis of SLE. Moreover, disease marker analysis of these 26 genes clearly showed that a series of more important genes (14/26) are directly related to SLE, including *CD180*, *MDM2*, *KLHDC7B*, *USP18*, *TLR8*, *DDX60*, *CD163*, *PXK*, *JAK2*, *GSTP1*, *CISH*, *GPX1*, *RGL1*, and *CCR1* (Figure 5C). In addition, the top scored network analyses indicated that three of the 26 selected genes, namely *USP18*, *GPX1*, and *RGL1*, were primarily enriched in transcription-related processes (translational initiation, and nuclear-transcribed mRNA catabolic process) (Figure 5D and Supplementary Table S6). These observations indicate that the important role of these three specific mRNAs with ac^4^C modification in lupus CD4^+^ T cells might be highly relevant to SLE by regulating mRNA catabolic processes and translational initiation. In summary, we assume that mRNA ac^4^C modification may function as a key factor in the pathogenesis of SLE.

## Discussion

As an emerging epigenetic field, epitranscriptome presents a powerful insight that diverse reversible posttranscriptional chemical modifications of mRNA are important regulators in human disease. Recently, great effort has been devoted to establishing the significance of the epitranscriptome in the regulation of eukaryotic gene expression, particularly in cancer and autoimmune diseases (O’Connell et al., 2015; Chen X. Y. et al., 2019). As recently reported, acetylation of cytidine has been identified as a mode of mRNA modification catalyzed by acetyltransferase NAT10, which enhances mRNA stability and elevates translational efficiency (Arango et al., 2018). However, the specific role of ac^4^C modifications in human disease is largely unknown. Therefore, we sought to explore the relationship between ac^4^C modification and SLE by performing epitranscriptome ac^4^C-modified profiles of lupus CD4^+^ T cells. As the proportion of CD4^+^ T cells in peripheral blood is small and epigenetics can vary from cell to cell, we pooled RNA of CD4^+^ T cells from 5/9 subjects, equally, into a single pool, to average the extremes and obtain a variety of profiles as described in previous study (Zhao et al., 2014; Guo et al., 2020).

Here, to the best of our knowledge, for the first time, we illustrated the new mRNA modification types in a global epitranscriptomic map of CD4^+^ T cells from SLE patients versus HCs, uncovering multiple modifications in CD4^+^ T cells of SLE patients that are distinctly different from those in CD4^+^ T cells of HCs. A significantly lower total ac^4^C level, as well as a decreased NAT10 expression level, was observed in the SLE group compared to HCs. NAT10 has been identified as the sole known human enzyme in eukaryotic cells to have both acetyltransferase and RNA binding activities, and which plays a crucial part in ribosome biogenesis (Sleiman and Dragon, 2019). Therefore, we speculated that the total ac^4^C level in SLE was primarily determined by the expression level of NAT10. However, we found a larger number of ac^4^C-modified transcripts in SLE than in HCs, by analyzing ac^4^C-RIP-Seq. The overall ac^4^C modification level of one sample is primarily determined by the numbers of ac^4^C modified transcripts in that sample, as well as the level of ac^4^C modification of each transcript. Considering the lower level of ac^4^C, and greater number of ac^4^C-modified transcripts in SLE patients, an increased number of ac^4^C-modified transcripts in one pool does not necessarily indicate an overall increased level of modified ac^4^C.

ac^4^C peaks are globally enriched within the CDS and 3′UTR regions of mRNAs in both HCs and SLE patients, which indicates that ac^4^C is a conserved internal transcriptional modification. However, ac^4^C sites in HeLa cells were observed proximal to translation start sites (5′UTRs and CDS region) in a previous study (Arango et al., 2018), which differs slightly from our observations. Similarly, differences in m^6^A peak distribution in different cells are evident in the following example. m^6^A peaks in osteosarcoma stem cells were mainly enriched in CDS, stop codon, and the beginning of 3′UTRs, whereas in clear cell renal cell carcinoma, they were enriched in the vicinity of transcription start site (TSS), CDS, and the stop codon (Wang et al., 2019; Chen et al., 2020). This was similar to what was observed in m^5^C sites in SLE patients, which were enriched in CDS and 3′UTR regions (Guo et al., 2020); however, the enrichment was primarily in the vicinity of the binding regions within 3′UTRs and regions immediately downstream of translation initiation in HeLa cells (Yang et al., 2017). Overall, different mRNA modifications are enriched in CDS region but have slightly biased distribution in 3′UTR, 5′UTR, and TSS regions, suggesting that the difference may be influenced by disease types, sample types, or sample sizes. Therefore, the localization of ac^4^C modifications in SLE may be a disease-related characteristic. Indeed, the top motifs identified for ac^4^C peaks, “CRGRA” and “CCRCCRC,” in lupus CD4^+^ T cells provide potential targets that differ from those in HCs. These specific motifs in SLE patients imply the potential presence of an as-yet unknown writer and reader, which needs to be validated in the future. However, ac^4^C peaks in HeLa cells present four obligate cytidines separated by two non-obligate nucleotides (CXX) (Arango et al., 2018), which in part is distinct from the patterns of ac^4^C modification in SLE. These differing results may be due to the different samples from different diseases, as well as the limitation of small sample size and heterogeneous sample pools in this study.

A total of 7952 differently ac^4^C-acetylated peaks were detected in SLE group compared to the HC group, including 3879 hyperacetylated and 4073 hypoacetylated peaks. Additionally, several differentially acetylated peaks were validated by ac^4^C-RIP-qPCR, which demonstrate the accuracy of our ac^4^C-RIP profiles. In contrast with the lower NAT10 expression in SLE patients, hyperacetylated peaks were observed in SLE patients. Similar to the methyltransferases of mRNA m^5^C modification in SLE, downregulated methyltransferase expressions along with aberrant mRNA m^5^C modification (hyper- and hypo-) were also found in SLE patients (Guo et al., 2020). Similar results have been obtained in bladder cancer, zebrafish, and PM2.5-induced pulmonary fibrosis mouse models (Chen X. Y. et al., 2019; Yang et al., 2019; Han et al., 2020). However, although an overall lower level of modified ac^4^C was observed in SLE patients, ac^4^C modified transcripts were not necessarily lower in any one sample. Moreover since it has been reported that several mRNA m^6^A methyltransferases, including METTL3/14, WTAP, and RBM15/15B, participate in installation of m^6^A modification in mRNA (He et al., 2019), we postulated that, in addition to NAT10, other “writer(s)” exist that regulate ac^4^C acetylation in SLE, accounting for the generation of more ac^4^C-peaks or hyperacetylated mRNA. Such differentially expressed ac^4^C-modified profiles might result from abnormal expression of multiple acetyltransferases. Bioinformatics analysis showed that these dys-acetylated peaks are involved in the regulation of cellular metabolism and several important immune and inflammatory pathways in lupus CD4^+^ T cells. The alterations in metabolism of CD4^+^ T involves regulation of normal immune responses (Choi et al., 2016); therefore, the metabolic processes associated with abnormally acetylated peaks in CD4^+^ T cells provide new insight into the molecular mechanisms of SLE. Among these inflammatory pathways, NF-κB signaling is involved in the transcription of numerous inflammatory genes and has been shown to be a molecular target in lupus T cells (Wong et al., 1999). Extensive research demonstrated fundamental roles for TGF-β receptor signaling in T cells contributing to autoimmunity (Morianos et al., 2019). Moreover, the top scored networks analysis showed that the hyperacetylated peaks participate in several central transcription processes, which has been verified in HeLa cells (Arango et al., 2018). These results imply that both hyperacetylated and hypoacetylated peaks are highly associated with lupus pathogenesis.

Previous reports have described a strong relationship between mRNA modification and gene expression (Dai et al., 2018; Chen X. Y. et al., 2019; Yang et al., 2019; Han et al., 2020), and hence, we sought to characterize the functional role of the ac^4^C modification in mRNA of lupus CD4^+^ T cells. Furthermore, as reported previously (Chen X. Y. et al., 2019), increased global mRNA m^5^C content in tumor samples relative to normal controls was identified by via LC-MC/MC analyses. The association of m^5^C modifications with gene expression was investigated and four different set of genes were found to be present. Chen X. Y. et al. (2019) further identified several oncogenes, which displayed both m^5^C hypermethylation and mRNA upregulation, and showed a significant correlation with bladder cancer. This phenomena has also been described in PM2.5-induced mouse pulmonary fibrosis (Han et al., 2020). Except for the ac^4^C modification, we could not delineate the integrated impact of other regulatory factors on the transcriptome profile in SLE, such as m^5^C and m^6^A modification (Chen et al., 2020; Guo et al., 2020). Moreover, as reported in our previous study (Guo et al., 2018, 2019), we found abnormal non-coding RNAs, such as miRNA and circRNAs, which serve as pivotal regulators in SLE, indicating that comprehensive factors contribute to the development of SLE. Intriguingly, large changes in RNA abundance were likely mediated by ac^4^C hyperacetylation or hypoacetylation in lupus CD4^+^ T cells. Taken together, ac^4^C modification may regulate expression of the modified upregulated mRNAs related to the pathogenesis of SLE.

Furthermore, the functional enrichment results of RNA-Seq combined with our previous data (Guo et al., 2018), suggest that a few dysregulated mRNAs are significantly enriched in the immune and inflammatory responses in either lupus PBMCs or CD4^+^ T cells, whereas certain mRNAs, which participate in NF-κB transcription factor activity, and antigen presentation by MHC II were found only in CD4^+^ T cells, parallel to the biological process of differently ac^4^C-acetylated peaks. The PPI network and GO analysis of all DEGs with ac^4^C modification indicate that they were involved in multiple essential processes. Therefore, we speculate that mRNA ac^4^C modification may be associated with pathogenic factors regulating SLE. Particularly, the GO analyses showed that only 26 specific hyper-up genes were associated with the pathogenesis of autoimmune diseases by regulating immune and inflammatory responses, and cytokine-mediated signaling pathways, which support the importance of ac^4^C modification in SLE. Moreover, we found that 14 specific genes out of 26 selected mRNAs are related to SLE, including *USP18*, *GPX1*, and *RGL1*. In light of the top scored network, 3 (*USP18*, *GPX1*, and *RGL1*) of the 26 selected mRNAs are intricately connected with the translational process. Therefore, we suspected that the three upregulated mRNAs with ac^4^C hyperacetylation in lupus CD4^+^ T cells were highly associated with SLE by regulating mRNA catabolic processes and translational initiation. Among the three common genes, *GPX1* has been reported in lupus endothelial dysfunction (Huang et al., 2020). *RGL1* has been identified as a novel target in inflammation-associated pathways of lymphocyte networks (Kirkby et al., 2014). In addition, *USP18* DNA are differentially methylated in interferon-regulated genes in lupus T cells, directly suggesting a mechanism for type I interferon hyper-responsiveness in SLE (Coit et al., 2013). Furthermore, the relationship between *USP18* DNA hypomethylated and *USP18* RNA ac^4^C hyperacetylated in SLE should be explored in the future. It has been reported that engineered gain of *USP18* decreases cancer growth by destabilizing growth-regulatory proteins (Mustachio et al., 2018), which suggests that *USP18* may alter transcribed mRNA catabolic process and translational initiation to facilitate the pathogenesis of SLE. In contrast, a previous study has discovered that the engineered loss of *USP18* increases ISGylation (François-Newton et al., 2011); therefore, upregulated *USP18* might reduce ISGylation to inactivate the interferon response, implying the function of ac^4^C modification in SLE which should be identified in further studies. However, the development of SLE depends on integrated modulation of various genes rather than a single gene. Hence, *USP18*, *GPX1*, and *RGL1* as they relate to SLE, may be potential ac^4^C-modified targets to explore the pathogenesis of SLE, which provides promising perspectives for additional study of ac^4^C-related pathogenic mechanisms. Although lower NAT10 expression directly resulted in lower levels of overall ac^4^C and hypoacetylated mRNAs in CD4^+^ T cells of SLE, we considered that other acetyltransferase(s) or integrated elements may also contribute to the hyperacetylated peaks. Based on the significance of GO analyses and a role of ac^4^C in the regulation of mRNA translation, it is necessary to further explore the mechanism of the 26 hyper-up mRNAs in SLE patients.

Taken together, these observations suggest that ac^4^C modification may participate in mRNA metabolism processing in SLE, leading to the activation of immune cells and inflammatory responses. In particular, mRNA ac^4^C modification might positively affect mRNA stabilization by upregulating certain SLE-related genes. Therefore, we consider that mRNA ac^4^C modification may act as an effective factor regulating the pathogenesis of SLE. However, certain limitations were noted in this study, including the highly heterogeneous sample pool, relative small sample size and lack of mRNA ac^4^C modification with SLE disease severity. Subsequent research using a larger sample size is needed to further validate the global ac^4^C alterations, and to explore the association of mRNA ac^4^C modification with the clinical outcome (disease severity) in SLE. As the most abundant, prevalent, and functionally relevant internal modification of RNA in eukaryotic cells, the regulatory function of m^6^A or m^5^C should also be jointly analyzed with ac^4^C modification in SLE. Additionally, association analyses between ac^4^C acetylation and related components (including length and GC content) would be essential for further understanding the biological function of ac^4^C acetylation. Above all, the impact of the specific ac^4^C-modified mRNAs on SLE pathogenesis should be investigated in future studies.

In summary, a transcriptome-wide ac^4^C modified panel of CD4^+^ T cells from HCs and SLE patients was illustrated to the best of our knowledge, for the first time, uncovering gene expression and SLE-related pathological pathways regulated by aberrated ac^4^C RNA modification. Our results provide potential proof of an association between mRNA ac^4^C modification and the SLE pathogenic mechanism, which should prove useful in prospective research to elucidate the mechanisms underlying ac^4^C modification in SLE. Moreover, the targeted genes of ac^4^C modifications are likely to serve as promising therapeutic targets for SLE.

## Data Availability Statement

The datasets generated for this study can be found in NCBI SRA accession PRJNA649884.

## Ethics Statement

This study was approved by the Medical Ethical Committees of the First Affiliated Hospital of Wenzhou Medical University (No. 2019121). All participants in this research provided written informed consent.

## Author Contributions

GG analyzed and interpreted the data. GG and HW drafted the manuscript. XS performed the experiments and statistical analysis. LY, XT, KY, ND, and CC acquired the data and provided material support. HZ and XX contributed to the conception and design of the study, analyzed and interpreted the data, supervised the study, provided the project funding, revised the manuscript, and finally approved the version of the manuscript for publication. All authors read and approved the final manuscript.

## Conflict of Interest

The authors declare that the research was conducted in the absence of any commercial or financial relationships that could be construed as a potential conflict of interest.

## Footnotes

1 https://portal.genego.com

2 http://metascape.org

3 http://david.abcc.ncifcrf.gov/

## Supplementary Material

FIGURE S1 Gene-specifc ac^4^C qPCR validation of ac^4^C level changes of five representative hypo-acetylated genes in SLE patients and HCs. **P* < 0.05; ***P* < 0.01.

FIGURE S2 Functional enrichment analysis of differentially expressed genes in CD4^+^ T cells from HCs and SLE patients. **(A)** Volcano plots display the differentially expressed genes with statistical significance (fold change > 1.5; *P* < 0.05). Red points indicate significantly upregulated genes and blue points indicate significantly downregulated genes. **(B)** Pathway maps and Process Networks analysis of DEGs.

FIGURE S3 Four quadrant graph shows the relationship between mRNA ac^4^C acetylated levels and its mRNA expressed levels. Yellow indicates upregulated DEGs with ac^4^C hypoacetylation (Hypo-Up), red indicates upregulated DEGs with ac^4^C hyperacetylation (Hyper-Up), green indicates downregulated DEGs with ac^4^C hypoacetylation (Hypo-Down), and blue indicates downregulated DEGs with ac^4^C hyperacetylation (Hyper-Down).

TABLE S1 Primers used for RT-qPCR and ac^4^C-RIP-qPCR.

TABLE S2 Most relevant networks.

TABLE S3 PPI of DEGs with ac^4^C Hyper or Hypo.

TABLE S4 Biological process and pathway categories of DEGs with ac^4^C hyper or hypo.

TABLE S5 Twenty-six specific genes correlated with autoimmune diseases.

TABLE S6 Most relevant networks for 26 autoimmune diseases related genes analyses.
