# Supplementary figures and images for: Epitranscriptomic N4-Acetylcytidine Profiling in CD4+ T Cells of Systemic Lupus Erythematosus
Source: Front Cell Dev Biol. 2020 Aug 28;8:842. doi: 10.3389/fcell.2020.00842 (PMC7483482; doi:10.3389/fcell.2020.00842)

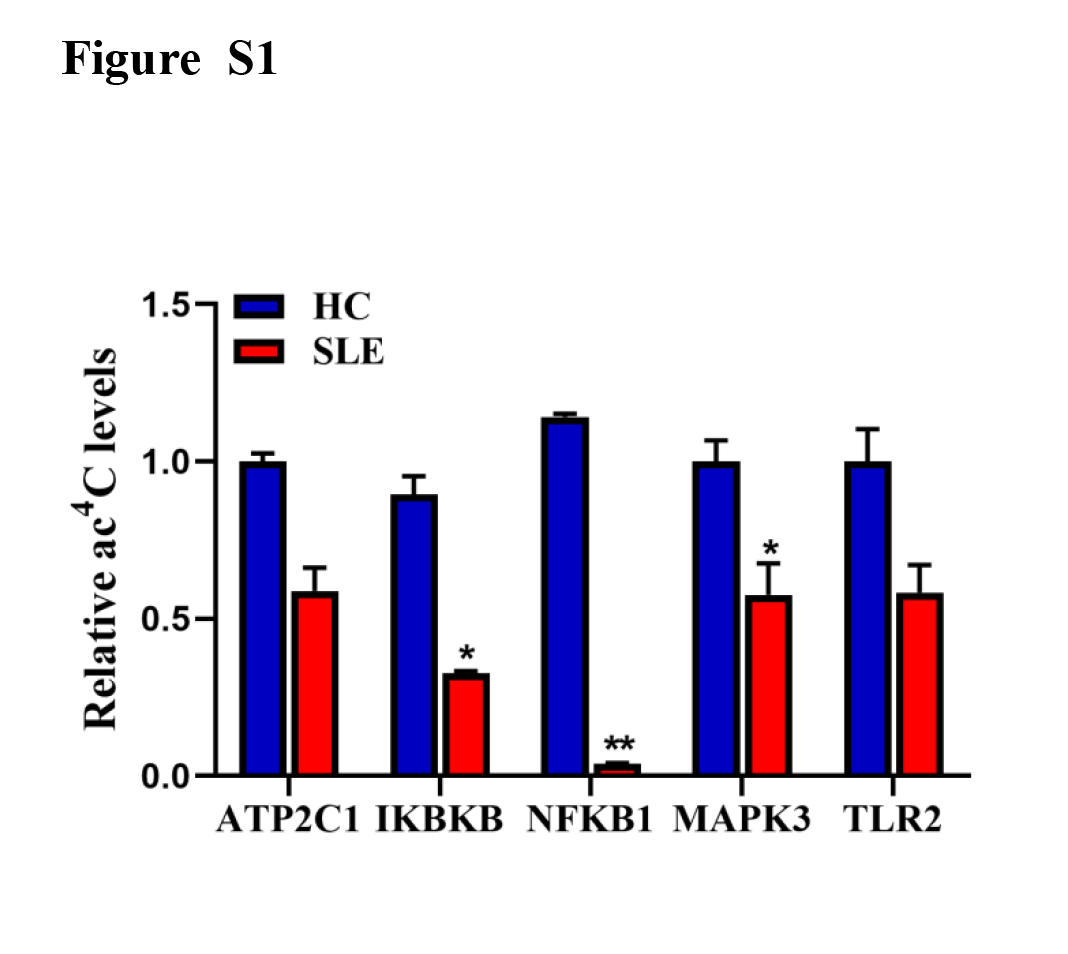

Supplement: FIGURE S1 — Gene-specifc ac4C qPCR validation of ac4C level changes of five representative hypo-acetylated genes in SLE patients and HCs. *P < 0.05; **P < 0.01. [file Image_1.TIF]

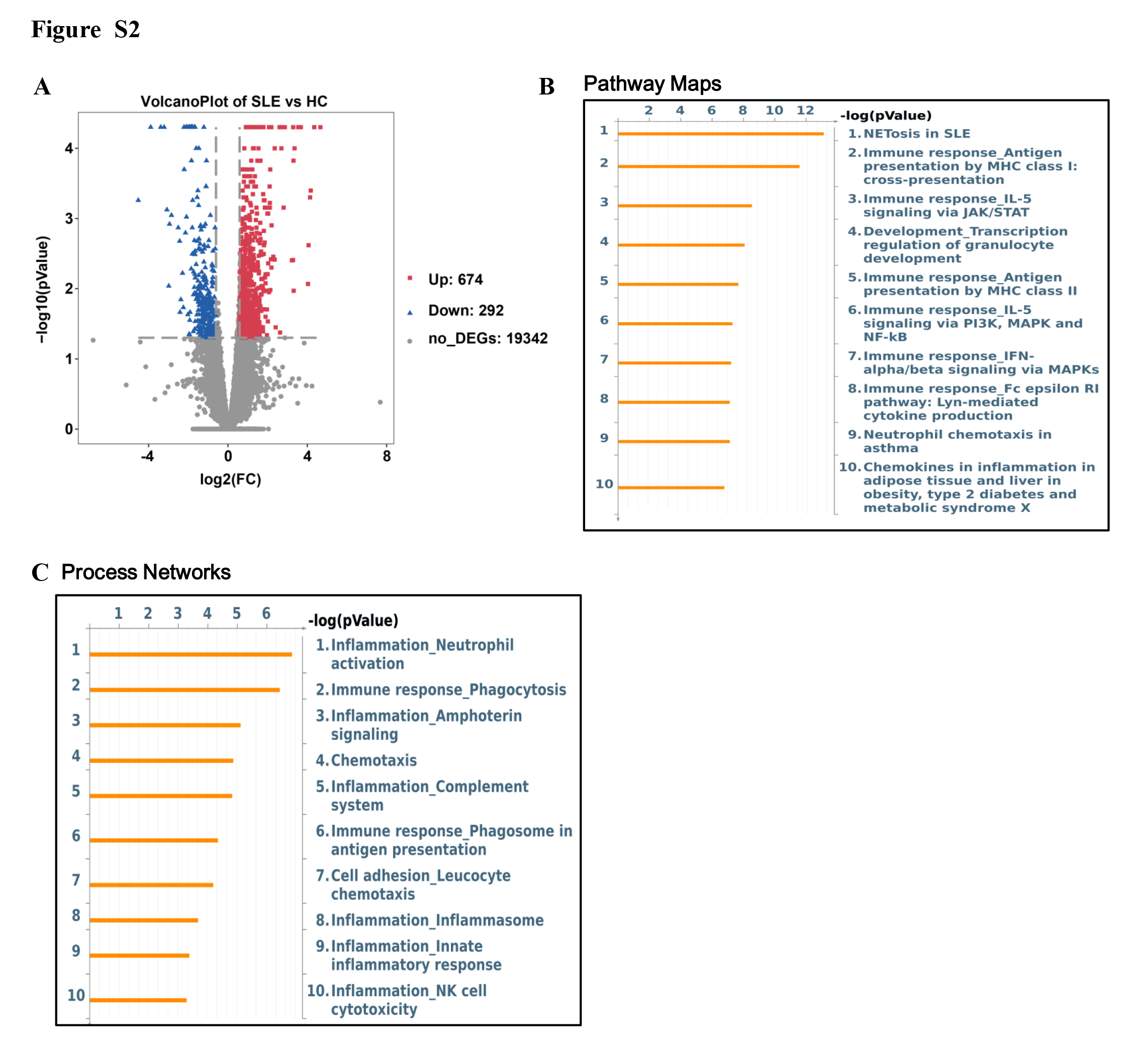

Supplement: FIGURE S2 — Functional enrichment analysis of differentially expressed genes in CD4+ T cells from HCs and SLE patients. (A) Volcano plots display the differentially expressed genes with statistical significance (fold change > 1.5; P < 0.05). Red points indicate significantly upregulated genes and blue points indicate significantly downregulated genes. (B) Pathway maps and Process Networks analysis of DEGs. [file Image_2.TIF]

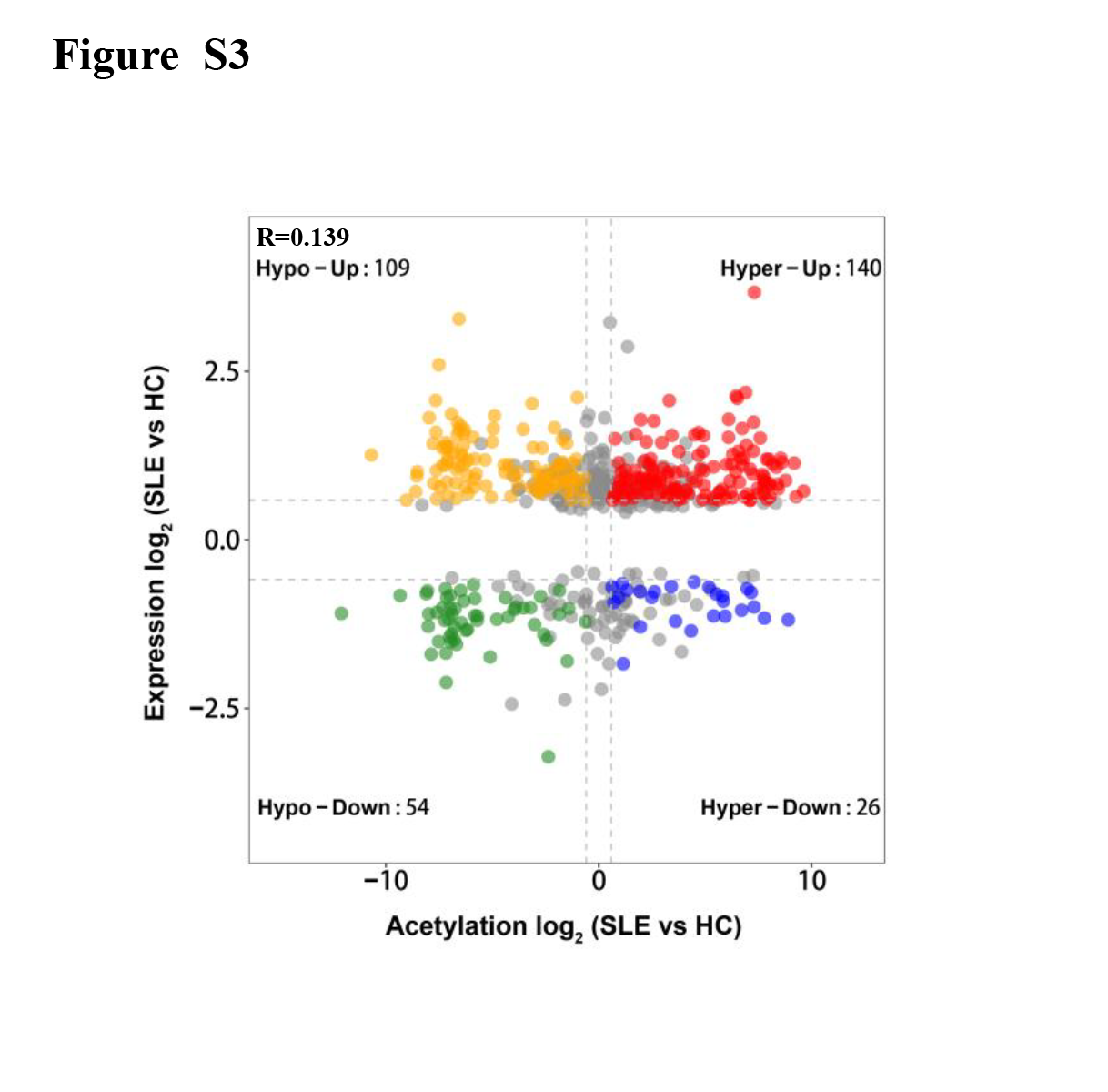

Supplement: FIGURE S3 — Four quadrant graph shows the relationship between mRNA ac4C acetylated levels and its mRNA expressed levels. Yellow indicates upregulated DEGs with ac4C hypoacetylation (Hypo-Up), red indicates upregulated DEGs with ac4C hyperacetylation (Hyper-Up), green indicates downregulated DEGs with ac4C hypoacetylation (Hypo-Down), and blue indicates downregulated DEGs with ac4C hyperacetylation (Hyper-Down). [file Image_3.TIF]
